# Fully integrated rapid microfluidic device translated from conventional 96-well ELISA kit

**DOI:** 10.1038/s41598-021-81433-y

**Published:** 2021-01-21

**Authors:** M. Jalal Uddin, Nabil H. Bhuiyan, Joon S. Shim

**Affiliations:** 1 Bio-IT Convergence Laboratory, Department of Electronics and Convergence Engineering, Kwangwoon University, Seoul, Republic of Korea; 2 Department of Electrical and Electronic Engineering, Islamic University, Kushtia, Bangladesh; 3 BioGeneSys Inc., Seoul, Republic of Korea

**Keywords:** Biological techniques, Engineering

## Abstract

In this work, a fully integrated active microfluidic device transforming a conventional 96-well kit into point-of-care testing (POCT) device was implemented to improve the performance of traditional enzyme-linked immunosorbent assay (ELISA). ELISA test by the conventional method often requires the collection of 96 samples for its operation as well as longer incubation time from hours to overnight, whereas our proposed device conducts ELISA immediately individualizing a 96-well for individual patients. To do that, a programmable and disposable on-chip pump and valve were integrated on the device for precise control and actuation of microfluidic reagents, which regulated a reaction time and reagent volume to support the optimized protocols of ELISA. Due to the on-chip pump and valve, ELISA could be executed with reduced consumption of reagents and shortening the assay time, which are crucial for conventional ELISA using 96-well microplate. To demonstrate highly sensitive detection and easy-to-use operation, this unconventional device was successfully applied for the quantification of cardiac troponin I (cTnI) of 4.88 pg/mL using a minimum sample volume of 30 µL with a shorter assay time of 15 min for each ELISA step. The limit of detection (LOD) thus obtained was significantly improved than the conventional 96-well platform.

## Introduction

Numerous infectious and immuno-related diseases such as malaria, tuberculosis, cardiac failure, and acquired immunodeficiency syndrome (AIDS) cause approximately fifteen million deaths worldwide every year^1,2^. More than 95% of these deaths take place in low-resource communities within developing countries because of a lack of cost-effective healthcare facilities to appropriately diagnose diseases and follow-up treatment^3,4^. As reliable diagnosis is an important step prior to initializing treatment, healthcare facilities have a critical impact in terms of making on-site clinical decisions. If diagnosis could be facilitated with cost-effective and easy-to-operate point-of-care (POC) rapid test kits, which provide accurate diagnoses without conventional laboratory equipment in resource-limited settings, this would save many lives.

For the last couple of decades, the enzyme-linked immunosorbent assay (ELISA) with a 96-well microplate and the paper-based lateral flow assay (LFA) have been widely used as advanced and reliable clinical diagnosis tools because of their specificity and sensitivity to antigen–antibody interactions^5,6^. In a conventional laboratory setup, ELISA is implemented with a 96-well microplate by a skilled technician and requires a large volume of expensive reagents, prolonged incubation time, and high-cost microplate reader^7–9^. In the case of conventional LFA however, the movement of the solution across the assay paper takes place through capillary action. Therefore, the reaction time cannot be precisely controlled, causing the non-specific binding of target analyte^10,11^. Since the viscosity of blood differs with age, the capillary-force initiated reaction time in the LFA varies from person to person. Thus, both the variation in reaction times and the issue of non-specific binding result in inaccurate point-of-care (POC) analysis. It is crucial that POC diagnosis should be fast and inexpensive with ease of execution, and thus convenient in resource-limited settings^12,13^. There is therefore a burgeoning interest in the implementation of conventional ELISA using a 96-well microplate and an LFA in the lab-on-a-chip (LOC) format in terms of on-site healthcare^14–17^.

Microfluidic technologies are extensively used to initiate the POC diagnosis of comprehensive health issues because of their unambiguous advantages^8,10,12^. Microfluidic devices may be integrated with cost-effective LOCs, along with various functional units such as PDMS micro pumps, valves, reactors^3,16,18^, and miniaturized analytical systems^19,20^. In addition, the microfluidic operation that employs micro-channels in a LOC device facilitates assay procedures, greatly reducing the assay time, together with the consumption of small amount of samples and reagents, without affecting specificity and sensitivity^21,22^. Therefore, an ELISA using a LOC device containing miniaturized structures may be implemented for use in POC diagnosis with optimized assay protocols^23^. Moreover, the majority of LOC devices can be fabricated using comparatively low-cost materials such as silicone, glass, and polymers that include polydimethylsiloxane (PDMS), poly(methylmethacrylate) (PMMA), and polycarbonates (PC)^24,25^, which initiate ELISA in a disposable LOC format^26,27^.

This paper presents a pump and valve-controlled LOC device converting a commercial 96-well microplate into a 96-well associated LOC device to execute an unconventional sandwich ELISA protocol intended to outweigh the negative aspects of conventional ELISA. In the conversion of a commercial 96-well microplate into a 96-well associated LOC device, the device integrates the dimension of a 96-well plate as an individual well onto a cost-effective PDMS micro-pillar that is fixed with a thermoplastic chip. The hexagonal well on the pillar is in contact within the 96-well plate, enabling a reaction zone for ELISA, and the PDMS pump and valve associated with the thermoplastic chip loads the sample and reagents into the reaction zone to conduct ELISA. The association of conventional 96-well plate with LOC counterparts expedites certain unique advantages over the 96-well microplate-based traditional ELISA, such as (i) a tinier reaction chamber inside the 96-well plate that enables ELISA with significantly reduced sample and reagent usage, and assay time compared to conventional 96-well microplate-based ELISA; (ii) the integration of the PDMS pump and valve precisely controls the flow rate addressing the imprecise reaction times of the different assay steps; (iii) as the reaction zone is comparatively small, the non-specific binding of target analyte may be effectively avoided by simple washing steps; and more importantly (iv) the ELISA can be conducted immediately for urgent patients as a POC format separating the 96-well from the entire cartridge.

The principle of a sandwich ELISA implemented using the proposed 96-well associated LOC device relies on the capture of antibodies and target antigen interaction, followed by the application of enzyme-linked detection antibody^28–30^. The further addition of a chromogenic substrate, which when catalyzed by the enzyme develops color in the detection zone, indicates the presence of the analyte of interest. The colorimetric post-ELISA images of the detection area are analyzed as relative gray scale value (GSV) using a dedicated image-processing android app. In a color image, each pixel corresponds to a luminance value regardless of its color indicating the brightness or intensity of the image, which is maximal for white color and minimal for black. Since white color has the components of red (r), green (g), and blue (b), the analyzed GSV is higher for a white image in comparison to its colored counterpart. To process the image analysis, the app selects a region-of-interest (ROI) both on the reaction zone and reference zone beside the reaction zone and calculates the GSV of each pixel in the selected regions. Finally, the relative GSV of the post-assayed image is determined by subtracting the averaged GSVs of the reaction zone from the reference GSVs.

### Design and operational principle behind the microfluidic ELISA device

**Scheme 1:**
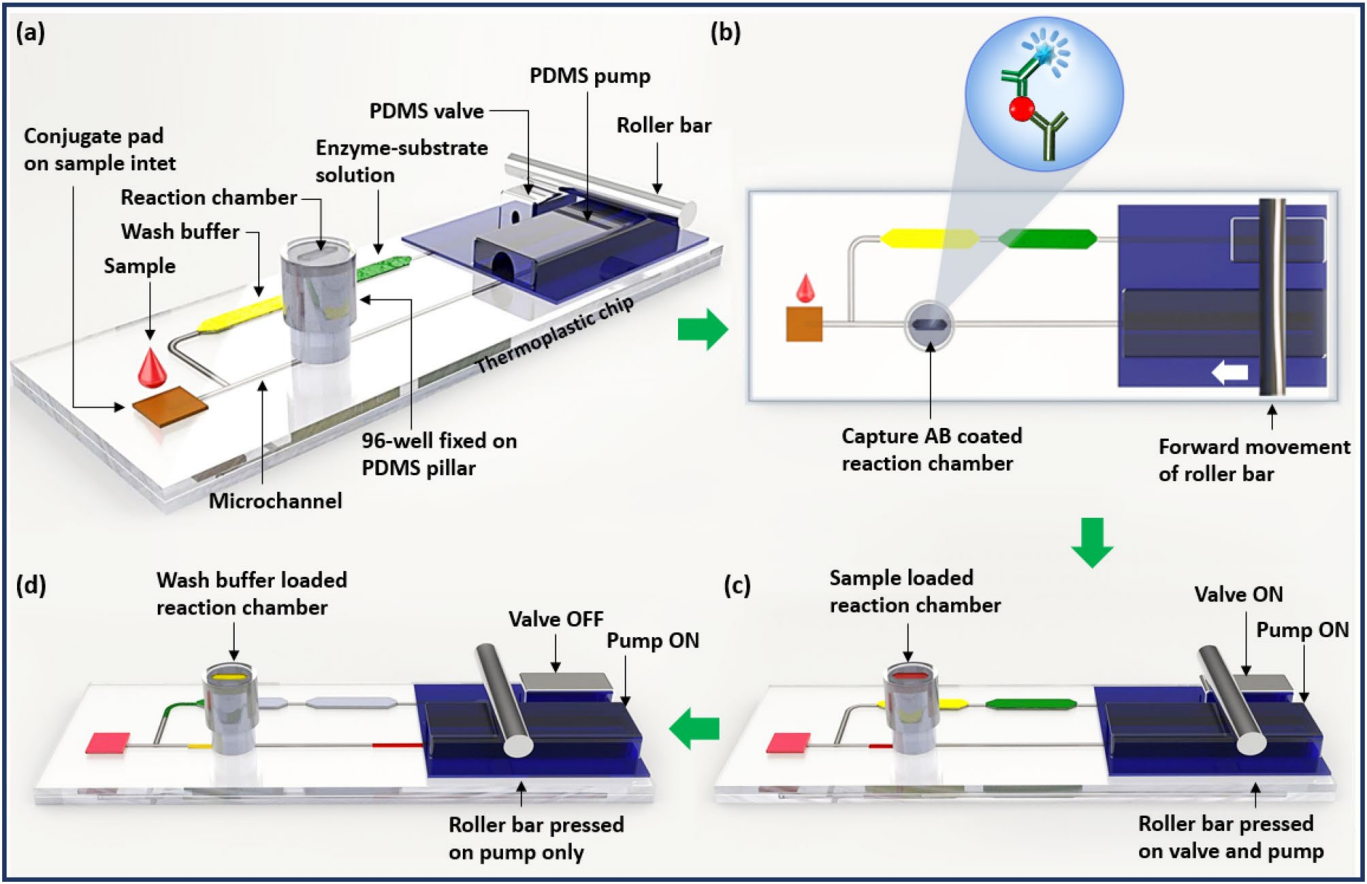
Conceptual illustration of the device. (**a**) A single 96-well microplate is fixed on a PDMS micropillar, which is attached with a thermoplastic chip, (**b**) a schematic top view of the device illustrating the ELISA procedure that is conducted using the proposed device, (**c**) pumping of the only sample into the reaction chamber when the valve is closed, and (**d**) pumping of wash buffer and enzyme–substrate solution into the reaction chamber, sequentially when the valve is open.

The proposed device (Scheme 1) includes (i) a PDMS pillar with a hexagonal reaction zone on top for conducting ELISA; (ii) a commercially purchased 96-well microplate piece fixed on the PDMS pillar; (iii) a PDMS actuator, including a pump and valve, for loading the sample and reagents; (iv) a microfluidic thermoplastic chip made of poly(methyl methacrylate) (PMMA) for microfluidic operation; and (v) a microprocessor-controlled mechanical roller bar to activate the PDMS pump and valve for microfluidic operation.

The PDMS pillar with a hexagonal deep space on its top, micro-pump and valve are attached onto the thermoplastic chip via back-to-back through-hole connections (Scheme 1a). The fixation of a 96-well microplate onto the PDMS pillar, as shown in Scheme 1a, facilitates a reaction chamber for ELISA. The thermoplastic chip accommodates the inlet to the target sample and reagent chamber for loading wash buffer and enzyme–substrate solutions for ELISA. The micro-channels in the thermoplastic chip assist the fluidic movement from the sample inlet and reagent chamber to the reaction zone with the assistance of the PDMS valve and pump. A microprocessor-controlled roller bar moves over the PDMS valve and pump and selectively pressurizes these pump and valve to sequentially load solutions from the inlet and reagent chamber to the reaction zone. The operational procedures of the PDMS pump and valve are demonstrated in Fig. S1.

## Results

In a sandwich ELISA system, two antibodies that are specific to an antigen correlate between the antigen level and responsive substrate by forming an antibody/antigen/antibody sandwich. To detect the target antigen, the capture antibody is pre-coated on the surface of interest, and the application of the target antigen and labelled detection antibody leads to the formation of a detection antibody/antigen/capture antibody complex. The entire process is followed by several incubation and washing steps, as in the case of conventional ELISA. The further addition of a chromogenic substrate evolves the perceivable color, with an intensity that corresponds to the amount of antigen present in the sample. The conventional ELISA is either executed using 96-well microplates or lateral flow assay paper.

The proposed 96-well associated LOC platform adopts certain features of 96-well microplates and LFA to provide a sandwich ELISA system, and thus strategically functions as an alternative to the conventional sandwich ELISA. During the assaying process, a miniaturized reaction chamber was prepared by integrating a commercial 96-well plate with a PDMS pillar to reduce the consumption of reagent and sample volume. In addition, a detection antibody conjugated pad was placed on the sample inlet of the 96-well associated LOC to shorten the assay time. In this case, the detection antibody specific to the target antigen was conjugated on the conjugate pad, as is the case in the majority of paper-based assays. Then the proposed 96-well associated LOC executes the ELISA process in three assay steps: (i) the dispensing of sample solution onto the conjugate pad selectively binds the target antigen with the detection antibody and forms the antigen/detection antibody complex; (ii) the application of this antigen/detection antibody complex into the 96-well associated LOC reaction chamber then binds the antigen with the capture antibody, thereby forming a detection antibody/antigen/capture antibody sandwich; and (iii) further addition of chromogenic substrate generates perceivable color signal interacting with enzyme-linked detection antibody of the detection antibody/antigen/capture antibody sandwich completing this unconventional ELISA. Thus, the proposed assay technique combines novel characteristics with the features of conventional ELISA, which are executed in LFA and using 96-well microplates.

### Optimization of sample volume and assay time

Conventional ELISA requires the consumption of a large sample volume along with a long incubation period at each assay step, which required the preparation of a miniaturized reaction zone with a reduced sample volume to shorten the assay time. To optimize the sample volume and assay time for the developed device, ELISA was conducted as demonstrated (Fig. S2) (i) using different volumes of wash buffer (ranging from 30 to 80 µL) with 30 µL of sample diluent and 30 µL of TMB substrate solution, and (ii) under various reaction times in different assay steps with a fixed volume of cTnI sample and TMB substrate (30 µL of both 78 pg/mL cTnI sample and TMB substrate) with an optimized volume of wash buffer for the detection of cTnI biomarker, respectively.

**Figure 1:**
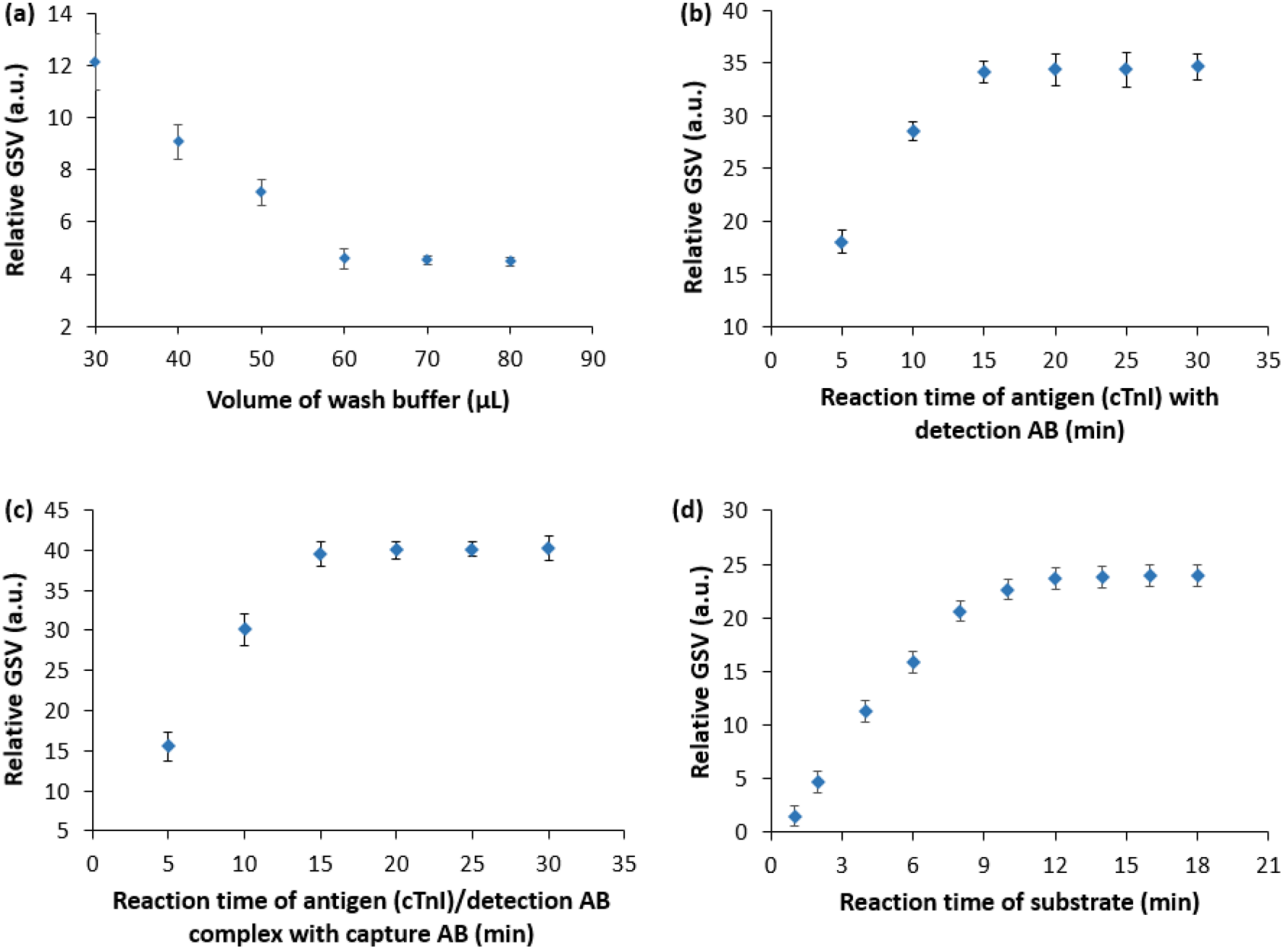
Optimization of (**a**) wash buffer volume and binding times for (**b**) the target antigen (cTnI) and detection antibody, (**c**) the target antigen (cTnI)/detection antibody complex and the capture antibody, and (**d**) TMB enzyme–substrate with capture antibody/antigen/detection antibody complex for the proposed ELISA protocol in the developed microfluidic platform. The error bars represent ± standard deviation of n = 3 samples.

Figure 1a shows the relative GSV for the differing volume of wash buffer solutions, whereas a comparative total of consumed wash buffer for each assay step both in the proposed and conventional 96-well based ELISA is shown in Fig. S2a. As seen (Fig. 1a), the relative GSV decreases with increasing wash buffer volume of until 60 µL and beyond that level, the relative GSV becomes stabilized. The increased relative GSV for the wash buffer in the range of 30–60 µL came up from the residuals of detection antibody and TMB molecules due to insufficient washing buffer. Beyond 60 µL the ELISA signal was reliably the same indicating the sufficient washing of residuals after every assay step and suggesting a minimum of 60 µL wash buffer or even more for ELISA using the proposed 96-well assisted LOC device. Moreover, the volume of wash buffer sufficient for each assay step in ELISA by the proposed device was significantly lower than its suggested volume in the conventional 96-well based ELISA (Fig. S3a).

To decide the optimized reaction times for the different assay steps, the binding time for both the antigen with the enzyme-linked detection antibody and the antigen/enzyme-linked detection antibody complex with the capture antibody were selected in the range of 5–30 min with some time spell, whereas the binding of TMB substrate with detection antibody/antigen/capture antibody complex was considered in the range of 2–18 min with 2 min time interval.

While ELISA was executed for various reaction times of the antigen with the enzyme-linked detection antibody, the reaction time of the antigen/enzyme-linked detection antibody complex with the capture antibody was maintained at 20 min and vice versa for binding of the antigen/enzyme-linked detection antibody complex with the capture antibody with 15 min binding time of TMB substrate with detection antibody/antigen/capture antibody complex for both the cases using the optimized volume of wash buffer. Then, based on their optimized reaction times and wash buffer volume, the binding time of TMB substrate with detection antibody/antigen/capture antibody complex was optimized. Figure 1b–d shows the optimized reaction time of different assay steps of ELISA using the 96-well associated LOC device.

Figure 1b shows the estimated relative GSV for the various reaction times of the antigen (cTnI) and detection antibody. The relative GSV was found to increase up to a reaction time of 15 min, beyond which the relative GSV was dependably steady. The relative GSV (Fig. 1c) is for varying reaction times of the antigen/detection antibody complex with the capture antibody, where the relative GSV also increased for the reaction time of up to 15 min and beyond 15 min, the relative GSV became saturated showing the similar trend (Fig. 1b). Moreover, the relative GSV (Fig. 1d) for the enzymatic substrate was stabilized after the reaction time of 12 min. Thus, a minimum reaction time of 15 min for the cTnI antigen with detection antibody, antigen/detection antibody complex with the capture antibody, and TMB substrate with detection antibody/antigen/capture antibody complex was obtained for the 96-well associated LOC ELISA device along with optimized wash buffer of 60 µL.

In the case in which the thermoplastic chip is reused, the tween 20 surfactant was loaded inside the microchannel for 5 min, then washed using DI water to avoid signal errors related to previous measurements. In quantifying the relative GSV, the area of the PDMS outside the reaction zone inside the 96-well microplate was recognized as the reference, and the difference between the GSVs of the reference area and reaction zone after an ELISA process was defined as the relative GSV of that cTnI level.

### Detailed ELISA using the proposed microfluidic platform

**Figure 2:**
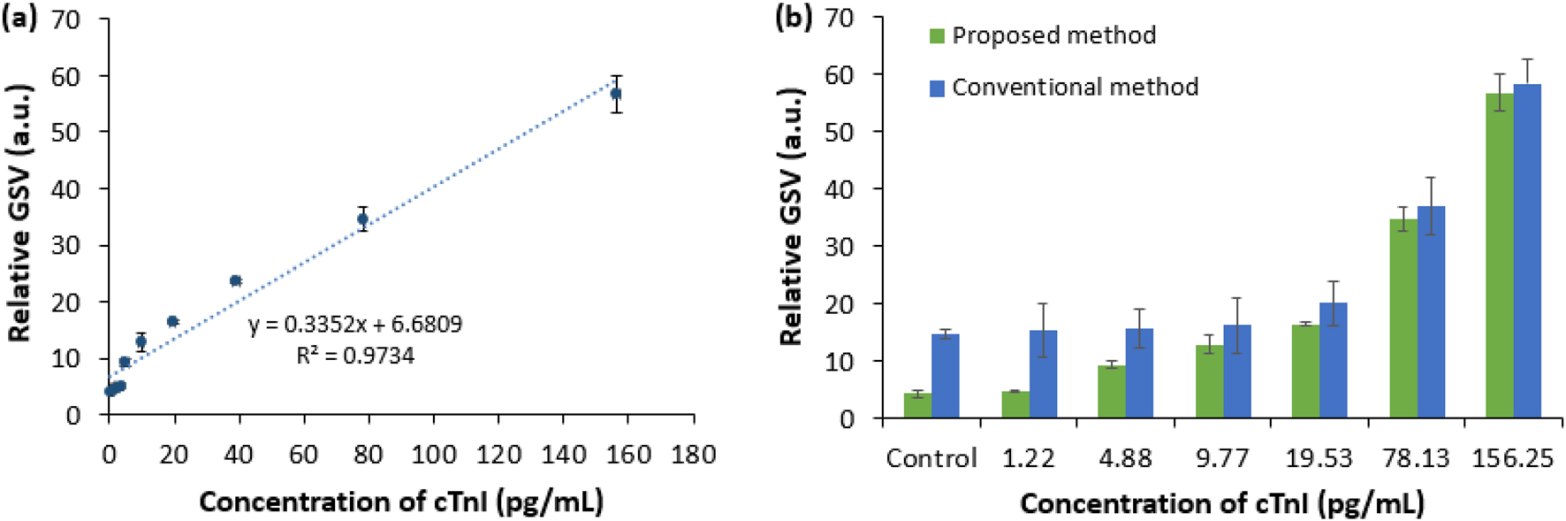
Relative GSV for (**a**) various concentrations of cTnI using ELISA procedures with the proposed microfluidic device, and (**b**) a comparison of the control signal and LOD of the proposed method with those of a conventional method, for six different levels of cTnI. The error bars represent ± standard deviation of n = 3 samples.

Based on the setup illustrated in the ‘Complete Assay Platform Preparation’ section later and the optimized assay time for the formation of the detection antibody/antigen complex, detection antibody/antigen/capture antibody sandwich, and enzymatic substrate described above, a detailed ELISA procedure was implemented to quantify the cTnI using the proposed LOC device. Figure 2a shows the detailed response of the ELISA procedure for increasing cTnI concentration. A comparison between a conventional 96-well and the proposed 96-well associated LOC ELISA device is shown in Fig. 2b.

From the correlation between the different concentrations of the cTnI and their corresponding relative GSVs (Fig. 2a), the relative GSV was found to be proportional to the concentration of the cTnI. As cTnI concentration increases, binding events between monoclonal capture antibodies and cTnI is enhanced. As a result, the linking of chromogenic TMB substrates with the detection antibody/ antigen/capture antibody complex increases, resulting in increased color intensity and increased relative GSV as well. The reference value obtained for the control signal was estimated in the absence of target antigen cTnI, and correlated with six levels of cTnI using both the conventional 96-well-based ELISA and our unconventional 96-well assisted LOC device (Fig. 2b). Both approaches exhibited a significantly increased signal with increasing cTnI concentration. The relative GSVs below 4.88 pg/mL and 19.53 pg/mL for the proposed and conventional approaches, respectively, were similar to that of the control sample. This indicates that the proposed device provides 400 percent (= 19.53 pg ml / 4.88 pg ml) improved limit of detection (LOD) compared to the conventional device, which is in the range of clinical threshold level^31,32^.

**Figure 3:**
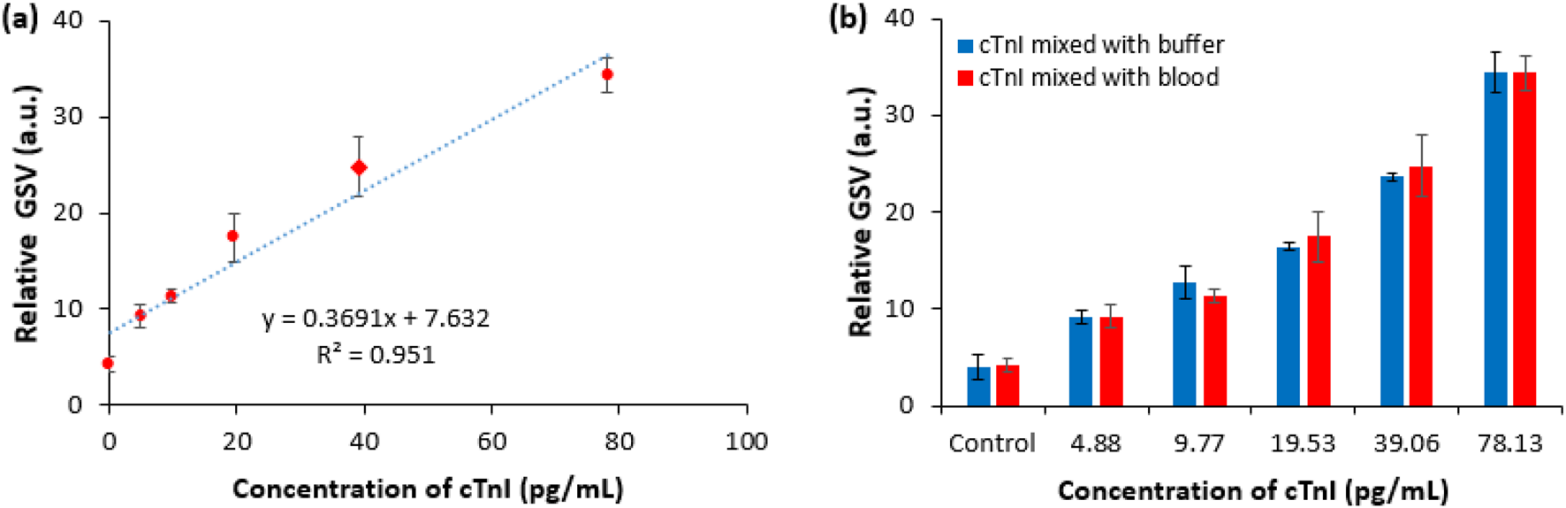
(**a**) Relative GSV for different concentrations of cTnI in blood with the developed device, and (**b**) comparative post-assayed relative GSV using the proposed ELISA device for different concentrations of cTnI mixed with buffer and diluted blood. The error bars represent ± standard deviation of n = 3 samples.

To validate the usefulness of the proposed device in the analysis of real blood sample for clinical applications, another study on detecting the different concentrations of cardiac biomarker (cTnI) was carried out following the ELISA protocol similar to that of various concentrations of cTnI prepared using buffer diluent reported in Fig. 2a. In the comparative case, different concentrations of cTnI (4.88, 9.77, 19.53, 39.06, 78.13 pg/mL) mixed with real blood sample were considered whose preparation has been explained in the “Materials and methods” section. Figure 3a shows the relative GSV for blood containing different levels of cTnI, whereas Fig. 3b shows the comparative relative GSV for cTnI mixed with blood and buffer. The relative GSV in Fig. 3a was proportional to the different levels of the cTnI in blood. Also, the relative GSV for cTnI mixed with blood and buffer diluent seems to be comparable (Fig. 3b) along with similar LOD implying the usefulness of the proposed device in reliable clinical applications.

## Discussion

The conventional ELISA protocols using a 96-well platform often requires collecting 96 samples for operation with a prolonged waiting time. Also, it requires expensive equipment in a laboratory setup and thus limits its application for POC diagnosis which is necessary for emergency rooms and small-size hospitals among others. Our device effectively resolves those issues of the conventional 96-well platform rapidly and cost-effectively. The proposed device individualizes the 96 well for individualized testing of ELISA using the conventional 96-well kit, which allows single patient testing at the earliest convenience. The integration of the disposable and non-disposable constituents in the device along with reduced consumption of reagents and samples assists in conducting ELISA very cost-effectively. Moreover, the LOD of the conventional 96-well platform is 400 percent (= 19.53 pg ml/4.88 pg ml) higher than our device. Thus, the sensitivity of our device is much better than the conventional 96-well platforms.

Since the implementation of the proposed unconventional method of ELISA was unworkable without the support of a 96-well plate and other reagents of commercial ELISA kit, the compatibility with LOC device in terms of the dimensional matching of PDMS pillar with 96-well plate was a major concern. As the capture antibody-coated surface of the 96-well plate in contact with the top of the PDMS pillar enables a compact reaction chamber, any dimensional mismatch could cause solution leakage resulting in inaccurate assay outcome. Moreover, the number of assay steps in an ELISA varies upon the binding method of a specific antibody with the target antigen following several washing phases and application of enzyme–substrate. To support the multistep ELISA, our proposed LOC device was carefully designed to accommodate the necessary on-chip sample inlet along with other reagent chambers. Thus, the device presented ensured compatibility with the commercially available ELISA kit.

In the proposed device, the sealing of the reagent chamber on-chip by Thermo Scientific Microplate Sealing Tape (Thermo Fisher Scientific Inc. USA) during each ELISA is laborious that could be addressed by further modification of the thermoplastic chip. In that case, the reagent chamber could be made as a closed reservoir along the channel inside the chip. The reagents (wash buffer and enzyme–substrate solution) could be loaded into the reservoir through two upper holes subsequently closing the holes by gently attaching Sealing Tape to it. When the roller bar moves over the PDMS actuator, the negative pressure originated inside the channel may keep the sealing tape attached with the surface of the chip, thus preventing any leakage during loading sample and reagents into the reaction chamber.

## Materials and methods

### Materials, sample, and conjugate pad preparation

The 96-well microplates, capture antibody, detector antibody, antibody diluent, cardiac Troponin I (cTnI) standard, sample diluent, enzyme–substrate (TMB), and concentrated wash buffer were purchased as commercially available ELISA kits from Abcam, UK. The lyophilized cTnI standard supplied with the ELISA kit was reconstituted using a sample diluent, and a stock solution of cTnI (50 ng/mL) was prepared by adding 100 µL of sample diluent to the standard. cTnI samples with a concentration of 1.22, 2.44, 4.88, 9.77, 19.53, 39.06, 78.13, 156.25, 312.50, and 625.00 pg/mL were then prepared through serial dilution of 50 ng/mL stock solution using a sample diluent at the ratio of 1:1. The second antibody cocktail of 1.5 mL was prepared by mixing 150 μL of the capture antibody and 150 μL of the detector antibody with 1.2 mL of the antibody diluent. The buffer wash was prepared by mixing deionized (DI) water with the concentrated wash buffer at a ratio of 9:1. A grade (319) sample pad and grade (8964) glass fiber-type conjugate pad were purchased from Boreda Biotech Co., Ltd. Kr. Tween 20 was purchased from Fisher Scientific, USA. The conjugate pad was cut into sections with dimensions of 0.5 cm × 0.5 cm, placed in the petri dish, and 50 μL of second antibody cocktail solution was dispensed onto each of the conjugate pad sections. The dispensed conjugate pad was then dried in a desiccator at room temperature, as previously reported^33^, and the closed petri dish was preserved in the refrigerator at 4–5 °C. To conduct ELISA using a real blood sample, the human blood was collected from LEE Biosolutions (USA) and then diluted at a dilution factor of 10 using the sample diluent (provided with ELISA kits from Abcam, UK). Then cTnI contained blood sample of 156.25 pg/mL concentration was prepared using the diluted blood and further samples of 78.13, 39.06, 19.53, 9.77, and 4.88 pg/mL of cTnI contained blood were prepared through serial dilution of 156.25 pg/mL solution using a sample diluent at the ratio of 1:1.

### Thermoplastic chip fabrication

The thermoplastic chip has dimensions of 12 cm × 3.2 cm and is comprised of two 2-mm thick PMMA layers. The top layer (Fig. S4a) includes a reagent chamber with dimensions of 45 mm × 0.55 mm × 2 mm, a sample inlet, and holes used to attach the PDMS pillar, pump, and valve. The bottom layer (Fig. S4b) contains a patterned microchannel of 0.5 mm width and 0.5 mm depth, for fluidic movement. The holes and micro-channels on the PMMA sheet were designed using the Corel Draw X8 program and patterned using a laser engraver (C30, Coryart Inc., Kr.) at a cutting speed of 11 mm/s at 30% power and a scan rate of 100 mm/s at 60% power. The two layers were then bonded together using a hot press at 90 °C for 15 min under an applied pressure of 2.75 MPa (Fig. S4c).

### Fabrication of PDMS pump, valve, and micropillar

The PDMS pump, valve, and micropillar are fabricated using the following steps: (i) the preparation of the PMMA molds is conducted to appropriately replicate the shape and size of the pump, valve, and pillar, and (ii) the designed patterns obtained using the PMMA molds are transferred to the physical structures using PDMS solution. To prepare the PDMS solution, the Sylgard 184 silicone elastomer curing agent and base (Dupont Inc., USA) were mixed homogeneously at a ratio of 1:14, and the mixture was placed inside a desiccator to remove air bubbles. The preparation of PMMA molds for the PDMS pump, valve, and pillar is illustrated (Figs. S5 and S6, respectively). To prepare the PDMS pump, valve, and pillar, the bubble-free PDMS solution was poured into the corresponding molds (Figs. S5a,c, and S6a), and cured at 80 °C for 30 min and 3 h, respectively. The PDMS pump (Fig. S5e) was made in two consecutive steps. First, the bubble-free PDMS solution of approximately 0.70 g was poured into the PMMA mold and cured at 80 °C for 10 min. The PDMS rod used for making the cavity in the pump was then placed in the rectangular hollow space of the mold, and the mold was fully filled with PDMS solution and cured at the same temperature for 20 min. The post-cured PDMS pump and valve were detached from the molds and the PMMA rod was removed from the PDMS pump to replicate the PDMS actuator for the pump and valve. Similarly, after detaching the post-cured PDMS pillar from the corresponding mold, the pins that were fixed in the mold (Fig. S6a), generates microfluidic channels in the PDMS pillar to load solution into the reaction zone from the sample inlet and reagent chamber and thus the PDMS pillar (Fig. S6b) using PDMS solution in the PMMA mold was replicated.

### Complete assay platform preparation

**Figure 4:**
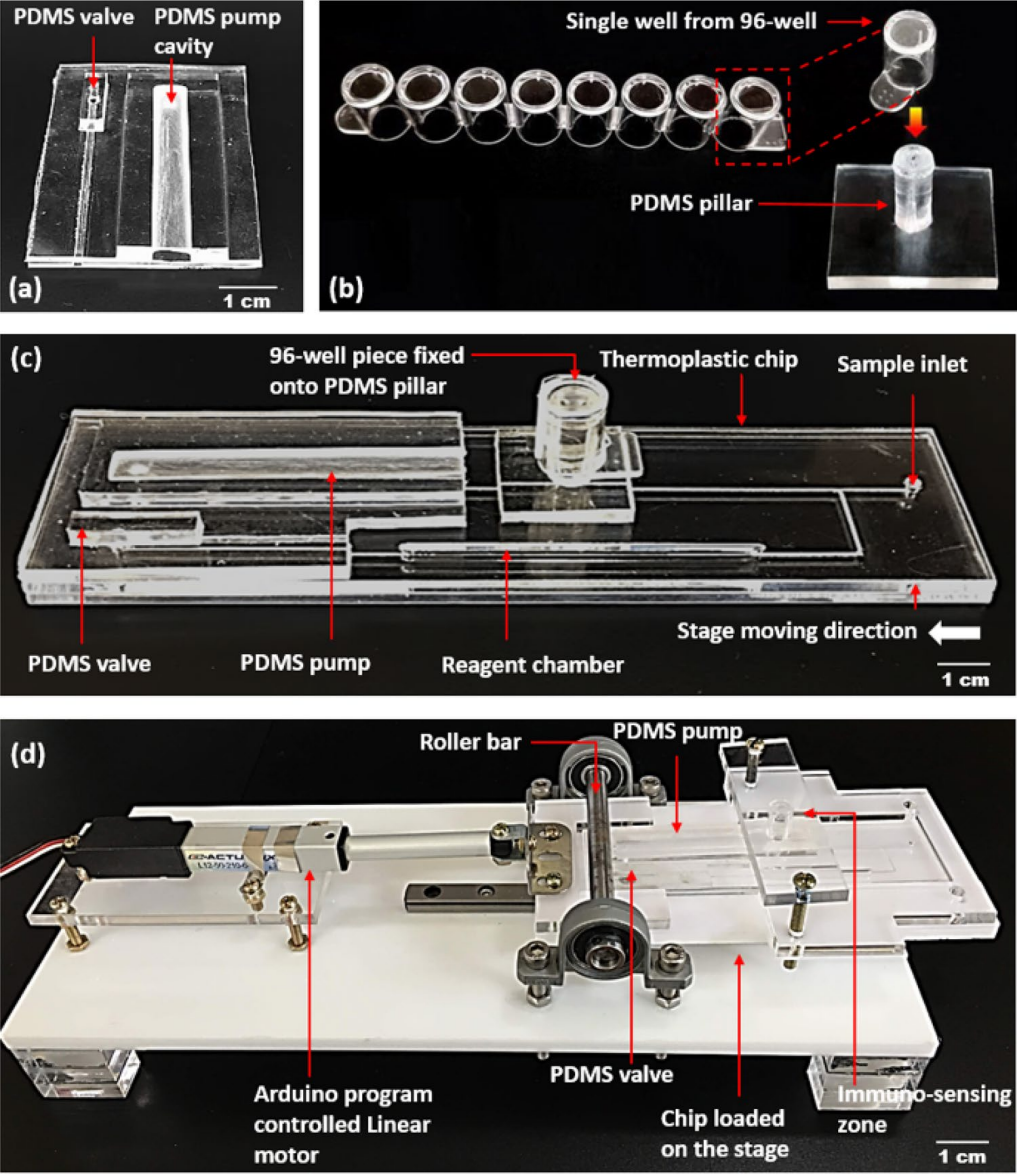
Details of the proposed device. The fabricated (**a**) PDMS actuator, (**b**) PDMS pillar to fix with a well separated from 96-well microplate. (**c**) The PDMS actuator and PDMS pillar attached to a thermoplastic LOC device with patterned micro-channels, and (**d**) the roller driving frame with a chip-loading stage is connected to a linear motor. The stage holding the thermoplastic chip moves laterally under a roller bar.

The entire immuno-sensing platform combines a PDMS actuator and a well separated from commercially purchased 96-well microplate with a PDMS pillar onto a thermoplastic chip. The entire chip is integrated with a linear motor driven (Firgelli Technologies Inc., Canada) chip loading stage. Details of the proposed device is shown in Fig. 4.

The PDMS actuator containing pump and valve (Fig. 4a), and PDMS micropillar (Fig. 4b) were attached to the thermoplastic chip using double-sided 3 M VHB Adhesive Tape purchased from 3 M Science, Kr. (Fig. 4c). While attached, the back-to-back positioning of through holes between the thermoplastic chip, PDMS pillar, pump, and valve were maintained to facilitate fluidic movement during the assay procedures. Finally, the thermoplastic chip with the pump, valve, and micropillar was then placed on the chip-loading stage of the roller driving frame (Fig. 4d). The movement of the stage that holds the thermoplastic chip under the roller bar was modulated using an external Arduino board that was connected with the linear motor. In the roller driving frame, the height of the roller bar was maintained at a specific level using bearings and screws. This ensured that the PDMS pump and valve are pressurized while the chip moves forward under the roller bar. For the assaying process, the single 96-well microplate that is vertically fixed on the PDMS pillar accommodates an immuno-sensing reaction zone at the top of the pillar, inside the 96-well. The working mechanism of the developed 96-well associated LOC microfluidic device is illustrated (Fig. S3) using dye solutions as the representative biomarkers and the entire process is demonstrated in Movie S1.

## Supplementary Information

Supplementary Information.

Supplementary Video 1.
