# Supplementary material for: Fully integrated rapid microfluidic device translated from conventional 96-well ELISA kit
Source: Sci Rep. 2021 Jan 21;11:1986. doi: 10.1038/s41598-021-81433-y (PMC7820004; doi:10.1038/s41598-021-81433-y)
Supplement: Supplementary file 1 — Supplementary Information. [file 41598_2021_81433_MOESM1_ESM.pdf]

Supporting Information (SI) for

Fully integrated rapid microfluidic device translated from conventional 96-well ELISA kit

M. Jalal Uddin[1,2], Nabil H. Bhuiyan[1] and J. Sub Shim[1]*

[1]Bio-IT Convergence Laboratory, Department of Electronics and Convergence Engineering, Kwangwoon University, Seoul, Republic of Korea.

[2]Department of Electrical and Electronic Engineering, Islamic University, Kushtia, Bangladesh

*Corresponding author: J. S. Shim Tel.: +82-10-4121-3075, E-mail: shim@kw.ac.kr

Operation of the PDMS pump and valve

Conceptually sketched top and corresponding cross-sectional views of the PDMS actuator are shown in Fig. S1a,b and Fig. S1a′,b′, respectively. This PDMS actuator used for loading target samples and reagents into the reaction zone is comprised of a micro-pump and a valve containing cavities. The pump and valve cavities are connected with the microchannels of the thermoplastic chip via back-to-back through holes. The pump cavity has dimensions of 0.5 cm (width), 0.2 cm (height) and 4.5 cm (length), whereas the valve cavity is much narrower, with a cross-sectional area of 0.785 mm^2 and a length of 1 cm. When a roller bar moves over the actuator and it is on both the pump and valve (Fig. S1a), both the pump and valve cavities are closed (Fig. S1a′). The pump generates negative pressure in the microchannel connecting the sample inlet to the reaction zone, whereas the valve blocks the microchannel connecting the reagent chamber to the reaction zone. Thus, the target sample is loaded into the reaction zone (Scheme 1a). When the roller bar

gradually moves on the PDMS actuator and remains only on the pump (Fig. S1b), the valve cavity is open (Fig. S1b′). The pump-initiated negative pressure is then active inside the microchannel connecting the wash buffer and enzyme substrate chamber to the reaction zone because the sample inlet is blocked by the sample's applied membrane filter. Consequently, the wash buffer and enzyme substrate solutions are sequentially loaded into the reaction zone by the gradual movement of the roller bar over the PDMS actuator (Scheme 1c,d).

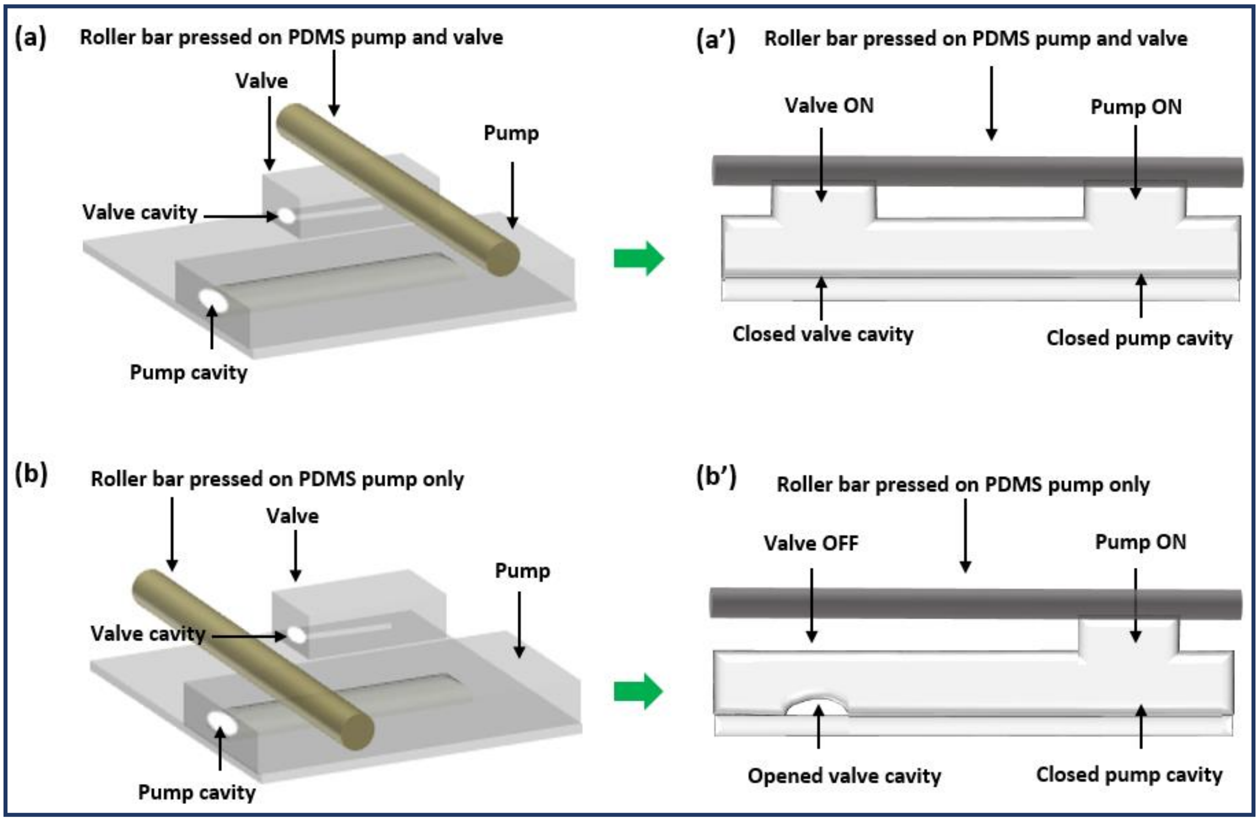

Figure S1. Conceptual top and cross-sectional view of the PDMS pump and valve. (**a**,**a**′) Roller bar pressurizing both the pump and valve bumper, and (**b**,**b**′) roller bar pressurizing only the pump bumper. Since the length of valve bumper is shorter than the pump bumper, the roller bar is just on the pump bumper with valve bumper OFF.

In executing ELISA, the proposed 96-well LOC platform should operate in two different phases in terms of the position of roller bar on the PDMS actuator. While loading the target sample into the reaction zone, the reagent-loaded chamber is sealed using tape, the detection antibody conjugate pad is placed on the sample inlet, and sample solution (red) is dropped onto the conjugate pad (Scheme 1a). In the first phase of the roller bar, it moves over the PDMS actuator covering both the pump and valve bumper. The microchannel connecting the reagent chamber to the reaction zone is blocked by the valve and forward movement of the roller bar originates negative pressure inside the channel and influxes the sample solution into the reaction zone via the conjugate pad (Scheme 1c). The application of sample solution on the conjugate pad thus closes the sample inlet as the porous of the conjugate pad is blocked by the sample solution. In the second phase of the roller bar, the bar moves further along. When the bar covers only the pump bumper of the PDMS actuator, the microchannel connecting the reagent chamber to the reaction zone opens, and the negative pressure inside the channel due to the forward movement of the bar loads the wash buffer (yellow) from the reagent chamber into the reaction zone (Scheme 1d). Finally, as the bar still covers only the pump bumper, the further movement of the roller bar washes the reaction chamber with the wash buffer and allows it to be filled by the green solution (enzymatic substrate).

Optimization of sample volume and assay time

In the comparative study on total of consumption wash buffer for each assay step both in the proposed and conventional 96-well based ELISA (Fig. S2a), the proposed platform requires 60 µL of wash buffer for each ELISA step which is almost seven and half times lesser than that of the conventional 96-well based ELISA. Also, Fig. S2b shows the total assay time required for the

proposed ELISA comparing with the conventional ELISA, where entire assay period for the conventional method is more than four and half times higher than that of the proposed 96-well LOC based ELISA. Thus, the proposed active microfluidic 96-well LOC enables ELISA with the consumption of relative smaller volume of sample and reagents and thus shortens the assay time.

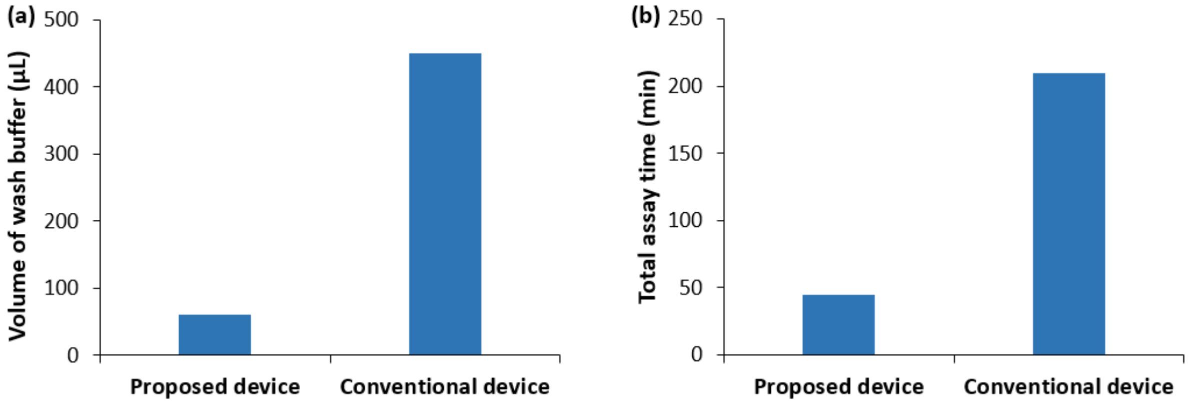

Figure S2. Comparative (**a**) total consumption of wash buffer, and (**b**) assay time for each assay step both in the proposed and conventional 96-well based ELISA.

Demonstration of the developed 96-well LOC platform for ELISA

There is a sample inlet, and a reagent chamber on the thermoplastic chip of the proposed platform. The sample inlet is used to load the target solution via the detection AB complex conjugate pad, whereas the reagent chamber assists the loading of wash buffer and enzymatic solution.

When the entire assay platform on the roller driving frame has been prepared using the process as explained; the reagent-loaded chamber of the thermoplastic chip was filled with 60 µL of red solution and 30 µL of green solution, and the chamber was closed using microplate sealing tape (Thermo Fisher Scientific Inc. USA) (Fig. S3a). The conjugate (reddish) pad with dimensions 0.5 cm x 0.5 cm was then placed on the sample inlet, followed by the placement of a sample pad with

a membrane filter (white) with dimensions 0.7 cm x 0.7 cm over the conjugate pad (Fig. S3b and S3c). Next, 30 µL of yellow target sample solution was dropped onto the sample pad (Fig. S3d), and the driving frame moved the PDMS actuator beneath the roller bar. Since the roller bar pressurizes the PDMS pump while moving, the sealed volume inside the microchannel of the thermoplastic chip deceases, thereby increasing the negative pressure. Thus, the negative pressure originating inside the channel results in the loading of the yellow sample solution into the reaction zone inside the 96-well microplate on the PDMS pillar, via the sample and conjugate pads (Fig. S3e).

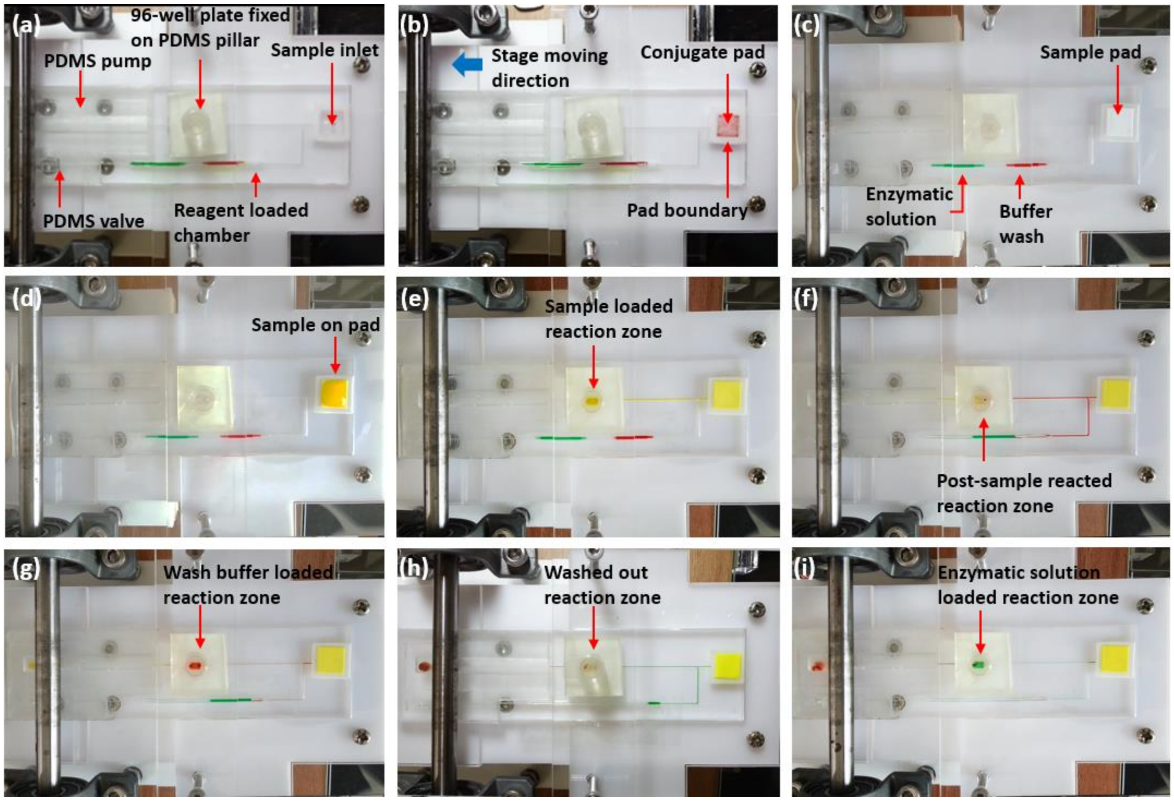

Figure S3**.** Microfluidic demonstration of the developed 96-well LOC platform for ELISA procedures using dye solutions. The demonstration follows these procedural stages: (**a**) fixation of red (wash buffer) and green (enzyme substrate) solutions in the loaded 96-well chip on the loading stage, (**b**) placement of enzyme-linked detection antibody conjugate pad (red) on the sample inlet,

(**c**) placement of filter membrane sample pad (white) on the conjugate pad, (**d**) dropping the target antigen (yellow solution) on the sample pad, **(e**) loading the target antigen (yellow solution) into the reaction chamber moving the PDMS actuator beneath the roller bar, (**f**) to (**h**) washing the reaction chamber using the wash buffer (red) by the movement of the PDMS actuator beneath the roller bar, and (**i**) loading the enzymatic substrate (green solution) into the reaction chamber by further movement of the PDMS actuator beneath the roller bar.

The chip gradually moved underneath the roller bar and immediately after the roller bar had passed over the valve bumper, the reagent-loaded channel was activated and wash buffer (red) from the reagent chamber was loaded into the reaction zone (Fig. S3f,g). In this case, the yellow solution fills the pores of the filter membrane and blocks the sample inlet. The further movement of the chip underneath the roller bar allows the enzymatic green substrate solution to be loaded into the washed out reagent chamber (Fig. S3h,i).

In real ELISA procedures, the reddish pad (Fig. S3b) corresponds to the conjugation of the enzyme-linked detection antibody onto the glass fiber conjugate pad. Furthermore, the yellow, red, and green solutions represent the target sample, wash buffer, and enzymatic substrate solutions, respectively. The detection zone is to be coated with the capture antibody to bind the target antigen in the sample and the loaded solutions should be retained into the reaction chamber based on the optimized reaction time required to successfully execute ELISA.

Fabrication of the thermoplastic chip

The detail layer-by-layer view of preparing thermoplastic chip is shown in Fig. S4. As seen, the device possesses two layers containing holes at the upper part (Fig. S4a) and microchannels at the lower part (Fig. S4b). The two layers are bonded together, as explained in Materials and method section of the main paper (Fig. S4c).

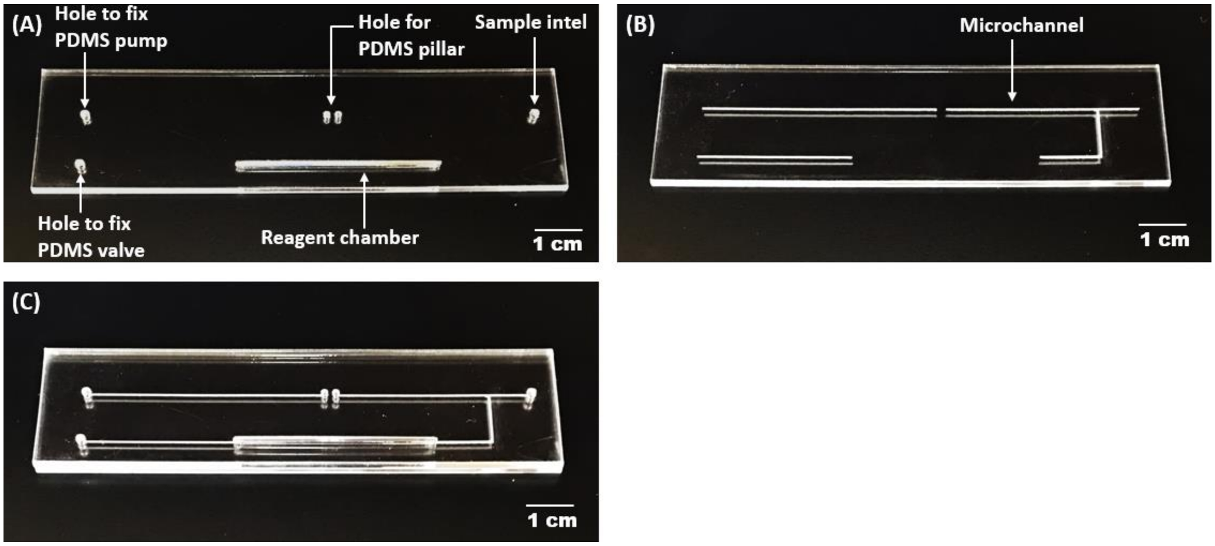

Figure S4. Layer-wise view of the (**a**) top, (**b**) bottom, and (**c**) two layers bonded together of the thermoplastic chip.

Fabrication of PMMA Mold for the PDMS pump, valve and micropillar

The fabrication of PMMA molds for the preparation of PDMS pump and valve are shown in Figs. S5a and S5c, respectively; whereas Fig. 6Sa corresponds to the fabrication of mold for PDMS pillar.

The PMMA molds for pump and valve (Fig. S5a and S5c) have three and two layers, respectively. The middle and bottom layer of the mold correspond to the dimension of 6.5 cm length x 5 cm width x 2 mm thickness, whereas its upper layer has the similar length and width along with 1 mm of thickness. The rectangular gap of 5.5 cm x 4.0 cm inner of the top layer replicates the base layer of the PDMS actuator, whereas the another two gaps of 5 cm x 1.5 cm and 1 cm x 0.4 cm dimensions replicate the bumper layers for the PDMS pump and valve, respectively. Finally, these three layers were bonded together (Fig. S5a) using hot press at 80 ^{o}C for 10 min under applied pressure of 2.5 MPa.

Both the two layers of the mold shown in Fig. S5c has the dimension similar to those of the top and bottom layers of the mold shown in Fig. S5a. These layers were also bonded together using the same bonding conditions. The dimension of the pump cavity was replicated using a PMMA rod of the dimension of 0.2 cm height, 4.5 cm width, and 4.7 cm length, whereas the replicated valve cavity was much narrower having cross-sectional area of 0.785 mm^2 and length of 1 cm. The PDMS layers (Fig. S5b and S5d) replicated using the molds (Fig. S5a and S5c), respectively were then bonded together using ozone treatment and thus, the fabricated PDMS pump and valve is shown in Fig. 53e.

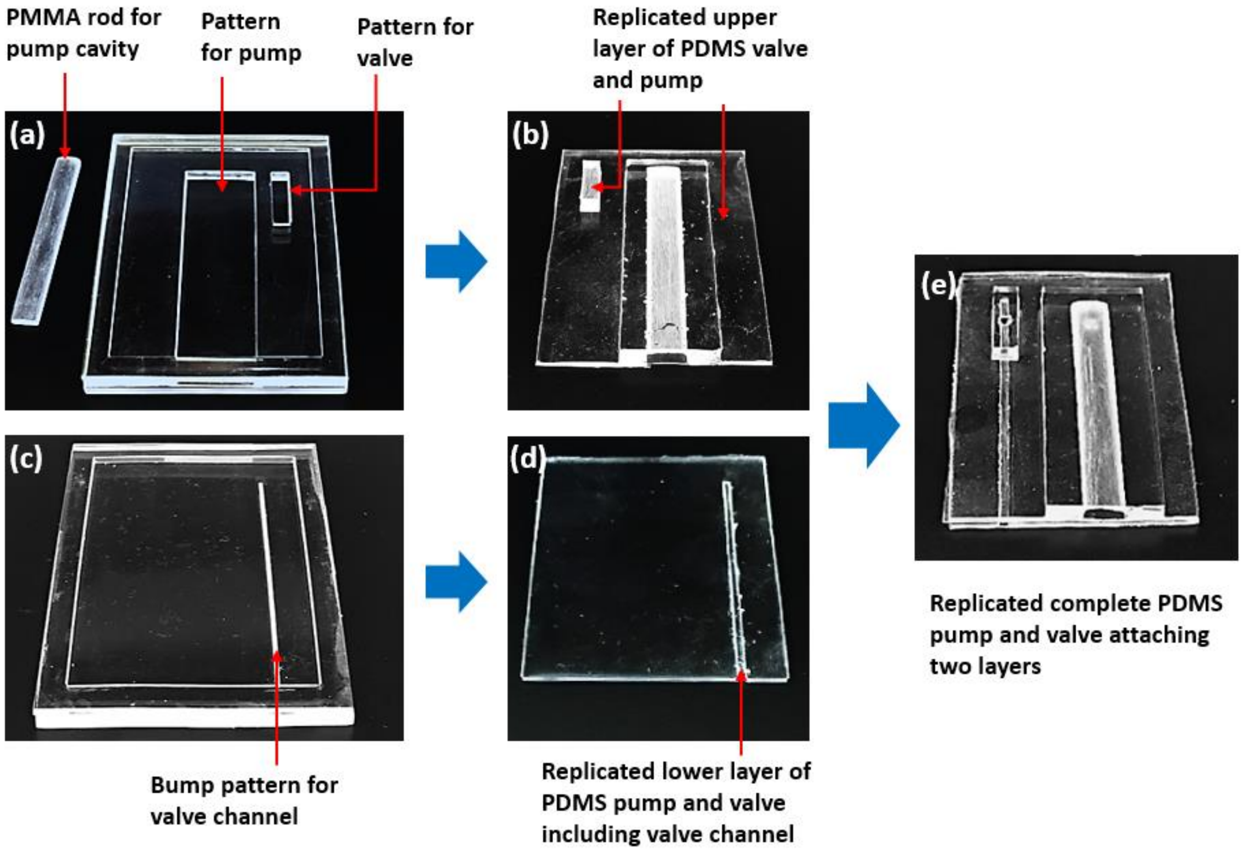

Figure S5. (a,c) PMMA molds for PDMS pump and valve, **(b,d)** replicated upper and lower layers of PDMS pump and valve, respectively **(e)** replicated entire PDMS pump and valve (with 1 cm scale bar for all the images).

Dimension of the different layer of the molds were maintained using the Corel Draw X8 program and the PMMA sheets were cut using a laser engraver (C30, Coryart Inc., Kr.) at a cutting speed of 11 mm/sec at 30% power and a scan rate of 100 mm/sec at 60% power.

Fig. 6Sa shows the mold having three PMMA layers for preparing PDMS pillar. All of these layers have 5 cm x 5 cm dimension with total thickness of 1.7 cm. The top layer of 2 mm thickness with 4 cm x 4 cm opening inside its boundary corresponds to the base layer for the PDMS micropillar, whereas the middle and bottom layers were engraved to replicate the shape and reaction chamber of the pillar using PDMS solution. The middle layer of 1.2 cm thickness has a hole of 5.8 mm diameter and the bottom layer of 3 mm thickness has a pattern of 2.3 mm x 5.5 mm x 1.5 mm dimension within a central circle of 5.8 mm diameter that replicates the reaction chamber on the PDMS pillar. Then, joining of the three layers together using 3M VHB Adhesive Tape (3M Science, Kr.) gives a complete shape of the mold (Fig. S6a) and the replicated PDMS pillar (Fig. S6b).

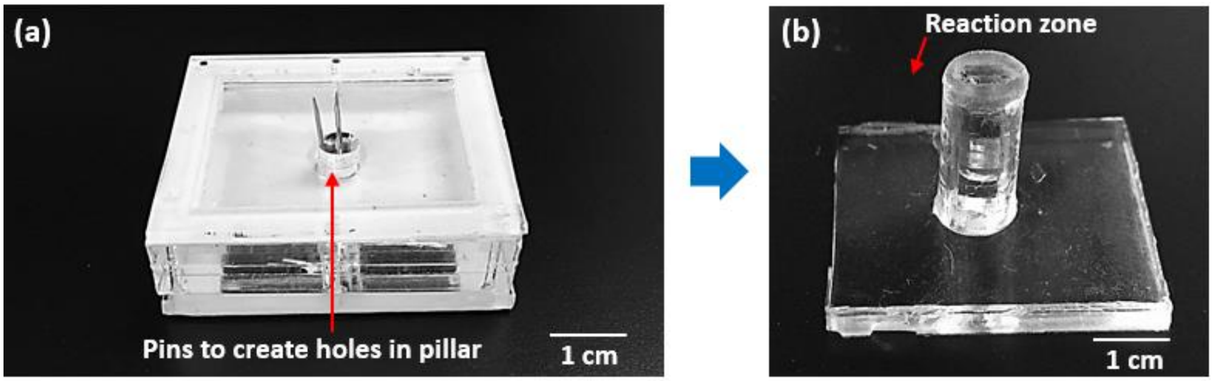

Figure S6. (a) PMMA mold for PDMS pillar, and (**b)** replicated PDMS pillar.
